# Malignant Dysentery

**Published:** 1852-11

**Authors:** Edwin R. Maxson

**Affiliations:** Adams Centre, Jefferson Co., N. Y.


ART. II.—*Malignant Dysentery.* By EDWIN R. MAXSON, M. D., Adams Centre, Jefferson Co., N. Y.

About the first of August, 1851, a dysentery, of a malignant character, made its appearance in our village and vicinity. The symptoms of which were as follows: The incipient stage was characterized by occasional nausea, and slight griping pains in the bowels; which continued from six to twenty-four hours. This was followed by a chill, more or less severe, with dysenteric discharges. The cold stage continued from one to twelve hours, during which the prostration was very considerable.

Then came the febrile reaction, which, in most cases, was very marked, and continued from seven to nine days; after which came fatal prostration, or gradual abatement of all the alarming symptoms, and slow convalescence.

During the twelve weeks it continued, I saw and treated about one hundred cases, more or less severe, from which I drew the following conclusions as to treatment. These conclusions have been confirmed by the result of numerous cases, of a similar character, occurring in the same locality this year. Cathartics, in every stage of the disease, were decidedly injurious, rendering mild cases desperate, and malignant ones fatal.

Of the five cases which I lost in 1851, three had taken either pills, salts, or oil, before I was called; and the other two, I gave small doses of Rhei, and Hydg. cum creta. Small doses of calomel, or even Hydg. cum creta, could not be borne without injury.

During the incipient stage, Tr. opii, gtt. xv, saturated with camph., given every three hours, did most toward arresting the disease.

After the chill, and during the cold stage, the same doses, with a teaspoon full of brandy, and sinapisms to the bowels, appeared to do best.

When the cold stage was succeeded by febrile reaction, with continued griping and frequent dysenteric discharges, I found the following to do well:

℞ Pulv. Doveri. gr. v.
Plumbi Acetas., gr. i.

Given every four hours.

After the febrile stage subsided, and the sweating commenced, there was generally considerable prostration; I then gave

℞ Pulv. Doveri, gr. v.
" Tannin, gr. ii.
" Camph. gr. i.

Every four or six hours.

All my cases were not treated precisely according to the preceding plan. But that was the general course, and the one which was the most successful.

Some of the cases were congestive; in these I gave, in addition, quinine. Others were of a typhoid character, some of which I blistered over the stomach and bowels, with very good effect.

Several circumstances, during the prevalence of the disease, suggested to me the possibility of its being somewhat contagious. A lady came from Watertown to see a friend, sick with the disease, and staid several days; after returning to her home, in the village of Watertown, where no disease of the kind was prevailing, she came down with it, became typhoid, and died.

Another lady, who was teaching in our vicinity, was exposed, and left for her home, thirty miles distant, in a healthy locality where no disease of the kind was prevailing; she too fell sick with the disease, and died. And then two of her sisters, who were living at home, and had not been otherwise exposed, took the disease and died.

It is common for us to have mild dysentery in August and September, and some bilious fever. But I know of no local cause that might be supposed to have produced this disease, except that very much vegetable matter was thrown up and exposed to the air, in a swamp a little to the west of us, in grading for the Watertown and Rome railroad during the spring and summer of 1841.

ADAMS CENTRE, N. Y., Sept. 24, 1852.

